# High-Resolution Estimation of Daily PM_2.5_ Levels in the Contiguous US Using Bi-LSTM with Attention

**DOI:** 10.3390/rs17010126

**Published:** 2025-01-02

**Authors:** Zhongying Wang, James L. Crooks, Elizabeth Anne Regan, Morteza Karimzadeh

**Affiliations:** 1 Department of Geography, University of Colorado Boulder, Boulder, CO 80302, USA; 2 National Jewish Health, Denver, CO 80206, USA; 3 Department of Epidemiology, Colorado School of Public Health, University of Colorado Anschutz Medical Campus, Aurora, CO 80045, USA

**Keywords:** air pollution, PM_2.5_, deep learning, spatiotemporal modeling, public dataset

## Abstract

Estimating surface-level PM_2.5_ concentrations at any given location is crucial for public health monitoring and cohort studies. Existing models and datasets for this purpose have limited precision, especially on high-concentration days. Additionally, due to the lack of open-source code, generating estimates for other areas and time periods remains cumbersome. We developed a novel deep learning-based model that improves the surface-level PM_2.5_ concentration estimates by capitalizing on the temporal dynamics of air quality. Specifically, we improve the estimation precision by developing a Long Short-Term Memory (LSTM) network with Attention and integrating multiple data sources, including in situ measurements, remotely sensed data, and wildfire smoke density observations, which improve the model’s ability to capture high-concentration events. We rigorously evaluate the model against existing products, demonstrating a 2.2% improvement in overall RMSE, and a 9.8% reduction in RMSE on high-concentration days, highlighting the superior performance of our approach, particularly on high-concentration days. Using the model, we have produced a comprehensive dataset of PM_2.5_ estimates from 2005 to 2021 for the contiguous United States and are releasing an open-source framework to ensure reproducibility and facilitate further adaptation in air quality studies.

## 1. Introduction

Air quality is a critical determinant of public health and environmental well-being, with particulate matter (PM) serving as a key indicator of pollution. Among PM components, particulate matter with a diameter of 2.5 μm or smaller (PM_2.5_) causes significant adverse health impacts [1–3] and environmental degradation [4].

PM_2.5_, originating from both natural sources, such as dust storms and forest fires, and anthropogenic sources, such as fossil fuel combustion and biomass burning [5]—as well as secondary formation from NO_2_, SO_2_, and NH_3_ emissions from vehicles, power plants, and agriculture [6]—can pose severe health risks due to their distribution into the terminal bronchioles and alveoli of the lung and potential systemic absorption [7,8]. Current well-calibrated air quality monitoring systems offer spatially and temporally coarse resolution surface-level measurements [9]. However, the uneven distribution of monitoring stations exacerbates the challenge of accurately estimating PM_2.5_ concentrations at high spatial resolution across large geographic areas. Despite the ongoing efforts to enhance air quality monitoring, significant challenges remain in comprehensively characterizing PM_2.5_ concentrations for use in, e.g., epidemiologic and clinical health studies. This limitation impedes our ability to effectively address emerging environmental and public health concerns [10,11].

Modeling methods for estimating surface-level PM_2.5_ concentrations encompass a range of techniques, including numerical model simulations; geostatistical interpolation approaches; land use regression (LUR) models; and, recently, statistical machine learning models. Each approach has distinct strengths and limitations. Numerical model simulations use mathematical equations to simulate the transport, transformation, and deposition of fine particulate matter in the atmosphere [12]. However, they are computationally intensive, especially for high-resolution spatial and temporal estimations over large geographies. This computation burden limits their practical application in large-scale studies [13]. Geostatistical interpolation approaches leverage spatial autocorrelation between monitoring data to generate continuous maps of PM_2.5_ concentrations. Their limitations include the assumption of spatial stationarity [14] and the potential for over-smoothing or underestimation of pollutant concentrations in areas with sparse monitoring sensor coverage [15]. LUR models predict the PM_2.5_ concentrations based on the relationship between land use variables (e.g., traffic density, land cover types, and proximity to emission sources) and observed pollution levels. Traditionally, LUR models suffer from low temporal resolution [16] and poor capability to capture non-linear effects, such as threshold and synergistic interactions among predictors [17]. However, recent advancements in spatiotemporal LUR models incorporate advanced statistical downscaling techniques that effectively capture spatial and temporal dependencies, thereby enhancing their precision in estimating PM_2.5_ concentrations across varied spatial and improving uncertainty quantification [18,19]. Machine learning approaches offer the flexibility to capture complex non-linear relationships between observed variables (e.g., meteorological data, land use characteristics, and remote sensing observation of aerosols) and observed surface-level PM_2.5_ concentrations. Moreover, these models excel in handling large datasets with high dimensionality, a critical advantage in the current era characterized by abundant remote sensing monitoring data and covariates. For instance, machine learning regression models like Gradient Boosting have been applied to forecast PM_2.5_ levels using air quality and meteorological data, demonstrating improved accuracy and robustness compared to traditional approaches [20]. Recent studies have applied various machine learning methods, particularly tree-based ensemble methods, to predict PM_2.5_ concentrations with enhanced accuracy [21,22].

Despite their success, these models follow a point-to-point estimation approach and do not fully leverage both spatial and temporal information, which further reduces performance, particularly in regions with heterogeneous pollution sources and complex atmospheric processes. Another critical gap in the current state of research is the limited consideration of wildfire impacts on PM_2.5_ levels, particularly during high-concentration days. Wildfires can significantly elevate PM_2.5_ concentrations due to the release of fine particulate matter from combustion processes [23]. Yet, many existing models fail to adequately account for this phenomenon, leading to poor performance in capturing extreme pollution events. Moreover, the temporal coverage and resolution of existing datasets often do not meet the requirements of cohort studies, and the lack of available open-source code makes it costly and challenging to generate estimations for new areas, times, or resolutions. These limitations present a major challenge for analysis of long-term trends and variations in air quality and their associated health impacts in cohort studies. For example, datasets like those presented by Di et al. [21] and Reid et al. [24] provide daily PM_2.5_ estimations from 2000 to 2015 for the contiguous U.S. and from 2008 to 2018 for 11 western U.S. states, respectively, and offer valuable insights into PM_2.5_ levels over certain periods and regions but do not extend to recent years.

Recently, Wei et al. [10] introduced a global daily PM_2.5_ dataset at 1 km resolution spanning from 2017 to 2022. The dataset is generated using an innovative 4D ensemble learning model by incorporating spatiotemporal autocorrelations, which improve accuracy in regions with sparse monitoring stations and heterogeneous pollution sources. While the dataset does not explicitly include wildfire-related variables, it evaluates performance during fire-affected periods, demonstrating its capacity to capture extreme pollution events such as those caused by wildfires and dust storms. Similarly, the LAPSO framework employs satellite observations, meteorological data, and deep forest (a cascade of random forests) to estimate near-surface pollutant concentrations [25]. The dataset is limited to the recent six years and lacks coverage of longer historical trends, which remain essential for long-term health and climate studies. Additionally, the focus is on creating a global model that offers extensive coverage rather than capturing finer-scale variations at the regional level.

This study aims to address the aforementioned research gaps within the overarching task of integrating satellite observations and ground measurements to generate a continuous surface with values estimating daily PM_2.5_ levels at 1 km resolution. We develop a novel model based on the latest advances in deep learning to better capture complex and nonlinear spatiotemporal relationships and generate more precise estimations. Specifically, our architecture is based on a Long Short-Term Memory (LSTM) network with Attention. To enhance the precision of estimates on high-concentration days, we incorporate wildfire smoke density into our modeling framework. We rigorously benchmark our model against the conventional widely used approach and also present quantitative comparisons of our data products against available datasets. Additionally, we make available a dataset of daily PM_2.5_ estimates at 1 km resolution over the contiguous United States spanning 25 August 2005 to 31 December 2021. This dataset is complemented by our open-source code and models, allowing estimation for other times and regions and promoting reproducibility and further research in the field.

## 2. Materials and Methods

### 2.1. Data Collection

Our study area and time were constrained to the contiguous United States from 5 August 2005 to 31 December 2021. The start date was selected to align with the availability of the wildfire smoke density dataset, and the end date was determined by the requirements of a longitudinal cohort research study of which this work is a component. However, we share our code under an open license to enable estimation for other time periods after 2021 as well.

We obtain ground-based PM_2.5_ measurements from the United States Environmental Protection Agency (EPA)’s Air Quality System (AQS) to serve as the prediction target for our model, as well as an auxiliary input, as described below. While these measurements are direct observations, their coverage is influenced by the uneven placement and sparse spatial density of monitoring stations. These stations are at times situated in areas with higher socioeconomic status, potentially overlooking regions with lower socioeconomic status and minority populations [26,27]. More importantly, for cohort studies, pollutant concentrations at residence or work addresses are important to obtain, and the stations are typically not located at those addresses. We aim to bridge the gap by offering refined, high-resolution PM_2.5_ estimates that encompass and leverage both monitored and unmonitored areas across the contiguous U.S.

Table 1 outlines the predictor variables utilized in our temporal models, detailing their sources along with spatial and temporal resolutions.

We utilized a comprehensive set of predictor variables to enhance the precision and robustness of our PM_2.5_ estimation model. Among these, satellite-derived Aerosol Optical Depth (AOD) at two spectral bands (Blue at 0.47 μm and Green at 0.55 μm) was included from the Moderate Resolution Imaging Spectroradiometer (MODIS) using the Multi-Angle Implementation of Atmospheric Correction algorithm (MAIAC), which is known for its high-resolution AOD retrievals [28]. However, the MAIAC AOD’s coverage is limited by factors such as cloud cover, orbit patterns, and the presence of snow or bright surfaces, which necessitated imputation to achieve gap-free AOD layers. We developed an XGBoost model for this purpose, detailed further in Appendix A.

Additionally, a set of meteorological variables from Daymet, including day length, precipitation, shortwave radiation, and minimum and maximum air temperatures, were used at 1 km spatial resolution and daily update frequency, providing essential environmental context [29]. Furthermore, wind direction and velocity data from gridMET at approximately 4 km spatial resolution were included to aid in modeling the transport and dispersion of air pollutants [30].

The National Oceanic and Atmospheric Administration’s (NOAA’s) Hazard Mapping System (HMS) Smoke product also played a crucial role by providing daily smoke density measurements from wildfires [31,32].

We captured vegetation dynamics using the 16-day Normalized Difference Vegetation Index (NDVI) from MODIS, assigning the closest available imagery to each estimation date. Additionally, we integrated elevation data from GMTED2010 to account for the impact of topography on air quality variation [33].

To effectively fuse satellite-observed data (i.e., the variables described above) with ground-measured PM_2.5_ levels, we generated another model input variable capturing proximal ground-based PM_2.5_ measurements using a K-Nearest Neighbor regressor with Inverse Distance Weighting (KNN-IDW). Specifically, for any given target location, KNN was used to identify the nine closest monitoring stations (based on geographic distance), and IDW was applied to assign weights inversely proportional to the distances between the stations and the target location, ensuring closer stations contributed more to the estimation. The choice of *k* (number of nearest neighbors) was determined based on exploratory experiments to balance overfitting and oversmoothing. A smaller *k* may lead to overfitting and boundary effects by over-relying on limited local observations, while a larger *k* may oversmooth and obscure local variations. We selected *k* = 9 as an optimal trade-off that captures local spatial variability while minimizing these drawbacks. Because our ultimate task is estimating surface-level concentrations from satellite observations (where ground-level measurements are missing), during the training of our deep learning model, ground-level measurements from the target location were intentionally excluded from the KNN-IDW calculations to prevent data leakage, thereby preserving the integrity of the validation process. In other words, for any given target location, satellite observations were fused with surface-level measurements only from nearby stations and not the surface-level measurements at the target location. After evaluation and to generate a continuous data product, all available data were incorporated to maximize the representativeness of surface measurements.

Additionally, latitude and longitude values were integrated to capture geographical variability in PM_2.5_ concentrations. This variability often stems from regional policies, economic activities, and specific geographic environmental factors, which can significantly influence air quality.

Temporal dynamics were also incorporated into our models. The day of the year and the month were encoded using cos and sin transformations to robustly capture seasonal variations in PM_2.5_ levels. These variations are pivotal to capturing weather conditions, variations in heating usage, and biogenic emissions throughout the year.

### 2.2. Model Architecture

The architecture of our model for estimating PM_2.5_ surface-level concentration employs a Bidirectional Long Short-Term Memory (Bi-LSTM) network, which is an enhancement of the LSTM architecture [34] designed to improve the learning of context in sequence prediction problems [35,36]. Unlike regular LSTMs, which process data in a unidirectional manner from past to present, Bi-LSTMs analyze the sequence both from past to present (forward) and present to past (backward), enhancing the model’s ability to assimilate contextual information from both pre-and post-event simultaneously [35]. The capability is vital for leveraging the temporal dynamics of environmental variables such as PM_2.5_, which are influenced by complex interactions of past conditions and immediate environmental factors. By incorporating both forward and backward data flows, the Bi-LSTM framework can better leverage irregular or regular patterns—such as weekly cyclical trends—that may be missed by unidirectional approaches, thereby providing a more robust prediction model for the dynamic and multifaceted nature of air quality monitoring.

We selected a 21-day input window for the model to balance training efficiency and predictive performance. While increasing the number of timesteps can enhance the model’s ability to capture longer-term temporal dependencies, it also increases computational complexity. A 21-day window allows the model to effectively capture weekly trends (e.g., within a three-week period) while minimizing training overhead. This selection ensures that the model remains computationally efficient without compromising its ability to leverage temporal patterns in the data.

**Figure 1.**
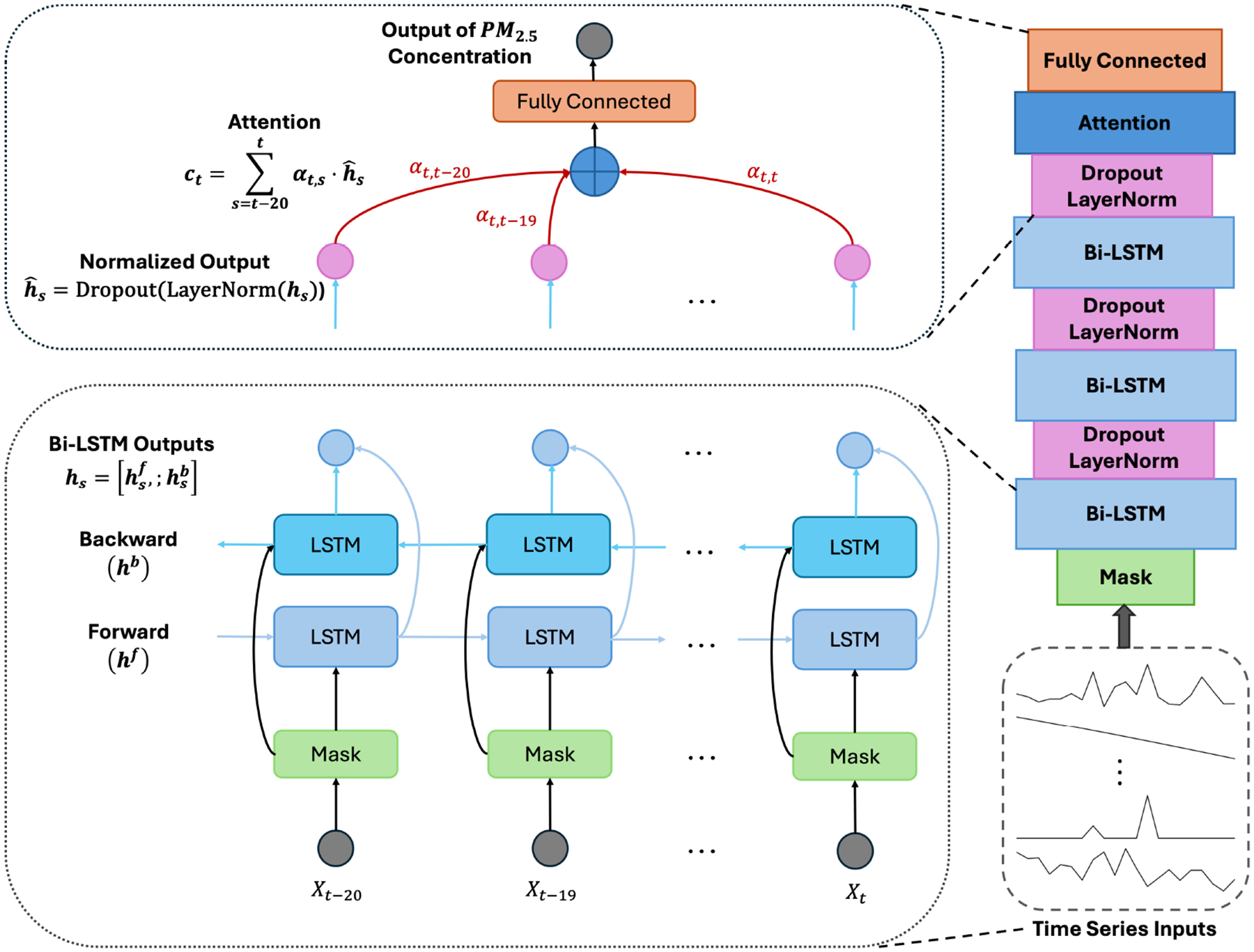
Model architecture of Bi-LSTM with attention mechanism. The architecture consists of three stacked Bi-LSTM layers that process input sequences in both forward and backward directions to capture temporal dependencies. An attention mechanism captures critical timesteps by assigning higher weights to Bi-LSTM outputs, and a fully connected layer generates the final PM_2.5_ predictions.

To handle missing data in the time-series inputs, we introduced a masking layer [37], enhancing the model’s ability to manage incomplete time-series information. Following each Bi-LSTM layer in our architecture (as illustrated in Figure 1), we applied layer normalization and dropout to stabilize the learning process and improve performance. Layer normalization optimizes training efficiency by normalizing inputs across the features for each sample rather than over the entire batch. Layer normalization ensures uniform scaling across neurons and helps mitigate internal covariate shifts [38]. This normalization is particularly beneficial in recurrent neural networks (RNNs) like ours, as it normalizes across the time-series sequence dimension and addresses issues associated with vanishing and exploding gradients [38]. Subsequently, a dropout layer is incorporated to prevent overfitting by randomly excluding a fraction (specifically, 20% in our model) of the features during each training iteration. This approach compels the network to learn more robust features that are effective in conjunction with various random subsets of other neurons, thereby enhancing the generalizability of the model.

In our Bi-LSTM model, we enhance time-series forecasting by integrating the Luong attention mechanism [39], which assigns dynamic importance to different timesteps (i.e., days) in the input sequence. Luong attention uses multiplicative attention instead of additive attention in Bahdanau attention [40], making it faster than Bahdanau attention. Based on our experiments, both attention mechanisms deliver comparable performance, but Luong attention is faster during model training. Each timestep *t* in the sequence is represented by a hidden state *h*_*t*_ of the Bi-LSTM layer that captures both forward and backward contextual information. Following the LSTM layer, layer normalization and dropout are applied to these states, resulting in normalized hidden states denoted as ${\hat{h}}_{t}$. Attention scores are computed using the dot product between the current normalized hidden state ${\hat{h}}_{t}$ and each prior normalized state ${\hat{h}}_{s}$ (for *s* ranging from *t* − 20 to *t*, corresponding to a 21-day input window), as formalized in Equation (1). The *W* matrix is a trainable parameter that helps determine the relevance of different parts of the input sequence by weighting the contributions of each state in the attention mechanism. These raw scores are then transformed into a normalized attention distribution using the softmax function, as described in Equation (2), such that all scores sum up to 1 across all hidden states.


(1) $$\text{score}\left({\hat{h}}_{t},{\hat{h}}_{s}\right)={\hat{h}}_{t}^{⊤}W{\hat{h}}_{s}$$

(2) $$\alpha_{t,s}=\frac{\text{exp}\left(\text{score}\left({\hat{h}}_{t},{\hat{h}}_{s}\right)\right)}{\sum_{s^{\prime }=t-20}^{t}\text{exp}\left(\text{score}\left({\hat{h}}_{t},{\hat{h}}_{s^{\prime }}\right)\right)}$$

The context vector *c*_*t*_, a weighted average of the hidden states, is then calculated using these weights, effectively summarizing the most informative aspects of the sequence up to *t*, as shown in Equation (3). This context vector is combined with the current state ${\hat{h}}_{t}$ to form the enhanced output state ${\tilde{h}}_{t}$, which is subsequently fed into a fully connected layer to generate the final prediction, depicted in Equation (4).


(3) $$c_{t}=\overset{t}{\underset{s=t-20}{\sum }}\alpha_{t,s}{\hat{h}}_{s}$$

(4) $${\tilde{h}}_{t}=\text{tanh}\left(W_{c}\left[c_{t};{\hat{h}}_{t}\right]\right)$$

This mechanism allows our model to dynamically focus on the most informative parts of the input sequence from both forward and backward directions, enhancing predictive performance, especially in the presence of complex temporal dynamics.

### 2.3. Model Training

To ensure the robustness of our model training, we normalized the inputs using a *MinMax Scaler* applied feature-wise, which scales each feature to a [−1, 1] range, facilitating faster convergence during training [41]. The architecture of our Bi-LSTM model includes three layers with progressively reduced neuron counts: the first layer contains 256 neurons, followed by two layers each with 128 neurons. This reduction in neuron count in subsequent layers acts as a form of regularization, helping to prevent overfitting while maintaining sufficient model complexity to capture underlying patterns in the data. This setting has been found to work best, according to multiple experiments. We employed an adaptive learning rate using an exponential decay scheduler, starting with an initial learning rate of 1*e*^−3^, and adjusting it at a decay rate of 0.8 every 30,000 steps. This approach, combined with the use of Adam optimizer [42], balances the benefits of momentum and adaptive learning rate adjustments [43], leading to efficient and stable convergence [42]. The model was trained with a batch size of 256 to maximize the utilization of an RTX 3080 GPU with 10 GB of memory. Furthermore, we selected Huber Loss as our loss function due to its robustness in handling outliers by combining the advantages of mean squared error and mean absolute error, providing a more stable training process [44]. This choice is particularly beneficial for time-series data, which can be susceptible to abrupt changes and noise [44].

### 2.4. Model Evaluation

For evaluating our PM_2.5_ estimation model, we adopted three primary metrics: *R*^2^, root mean squared error (RMSE), and mean bias error (MBE). *R*^2^ is calculated as $R_{2}=1-\frac{\sum_{i=1}^{h}{\left(y_{i}-{\hat{y}}_{i}\right)}^{2}}{\sum_{i=1}^{n}{\left(y_{i}-\bar{y}\right)}^{2}}$, where *y*_*i*_ represents the actual values, ${\hat{y}}_{i}$ are the predicted values, $\bar{y}$ is the mean of the actual values, and *n* is the number of observations. Root mean squared error (RMSE), formulated as $\text{RMSE}=\sqrt{\frac{\sum_{i=1}^{n}{\left(y_{i}-{\hat{y}}_{i}\right)}^{2}}{n}}$, quantifies the average magnitude of the prediction error. Mean bias error (MBE) is calculated by $MBE=\frac{\sum_{i=1}^{n}\left({\hat{y}}_{i}-y_{i}\right)}{n}$ and assesses the average bias in the predictions, indicating whether the model systematically overestimates or underestimates the actual values.

Our validation strategy involved a dual approach: a 10% held-out test set (approximately 300,000 data points) was randomly selected to evaluate the final model performance, and 10-fold random cross-validation was applied to the remaining 90% of the data (approximately 2,430,000 for training set and 270,000 validation set) to ensure the robustness and reliability of the model across different subsets of data. The validation results reported are the average outcomes across the ten folds, while the testing results are derived from applying the best-performing model, as determined by cross-validation, to the held-out test set.

We also benchmarked our model against other model architectures and compared our data product with other available datasets. First, we compared our model performance against widely utilized models in the field of PM_2.5_ estimation: the random forest model. The random forest model is a traditional choice in environmental studies due to its efficacy in handling non-linear data relationships and feature interactions [21,24]. We optimized the hyperparameters of the comparison models using random search, ensuring a fair and rigorous evaluation. Lastly, we also compare our model estimations with the widely used ensemble-based PM_2.5_ estimations by Di et al. [21]. Their approach integrates multiple machine learning techniques to achieve high spatiotemporal resolution across the contiguous U.S., providing a rigorous comparison for our model.

## 3. Results

### 3.1. Comparison Against Strong Baseline Methods

Table 2 presents the 10-fold random cross-validation and independent testing results, comparing our Bi-LSTM model with attention to two strong baselines, one being a standard LSTM model with attention and the other a random forest baseline. Our Bi-LSTM model consistently demonstrates superior performance across both the validation and testing phases. Specifically, during validation, our model achieved the highest average *R*^2^ of 0.74, surpassing both the LSTM (0.72) and random forest (0.69). Additionally, it achieved the lowest RMSE at 3.67 μg/m^3^, compared to 3.77 μg/m^3^ for LSTM and 3.98 μg/m^3^ for random forest. Our model shows negligible underestimation with an MBE of −0.09 μg/m^3^, compared to the nearly unbiased MBE of 0.00 μg/m^3^ for random forest and the slightly higher underestimation of the standard LSTM (−0.11 μg/m^3^). In the testing phases, our Bi-LSTM model continued to demonstrate strong performance with an *R*^2^ of 0.75, better than LSTM’s 0.72 and random forest’s 0.68, and achieved the lowest testing RMSE of 3.69 μg/m^3^. The testing MBE of our model was −0.08 μg/m^3^, further exhibiting a slight underestimation. However, this represents an improvement compared to the standard LSTM, highlighting the effectiveness of Bi-LSTM’s bidirectional architecture in reducing bias during PM_2.5_ estimation.

**Figure 2.**
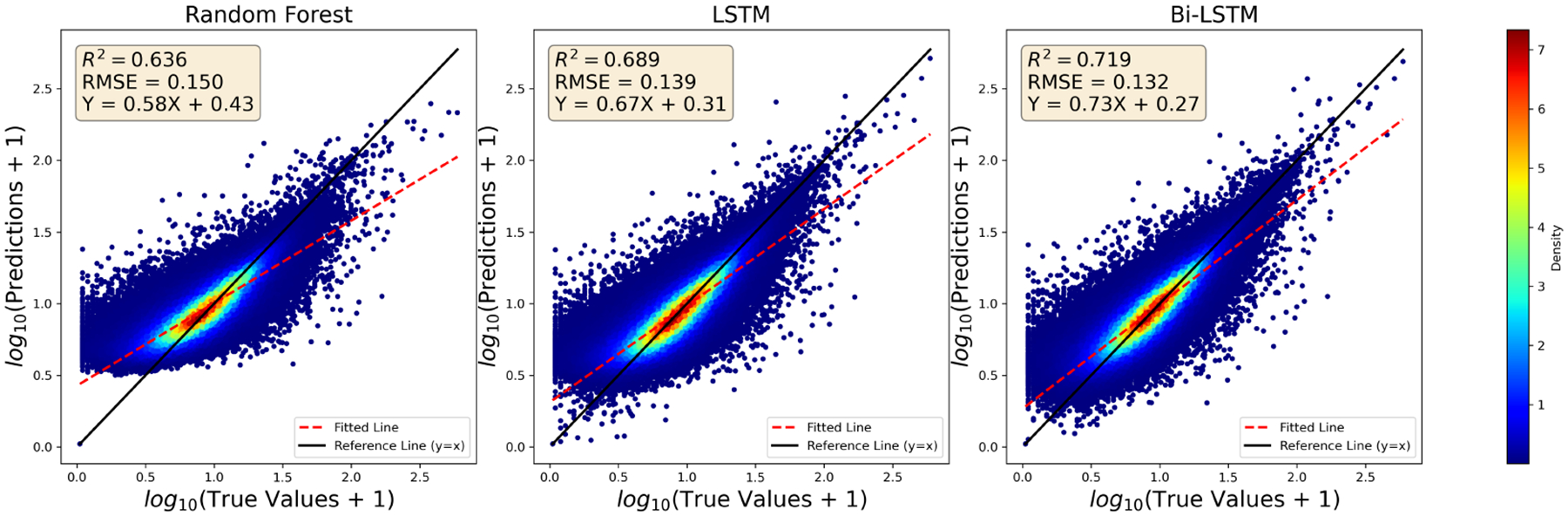
Density scatter plots comparing PM_2.5_ predictions versus true values for random forest, LSTM, and Bi-LSTM with Attention Models. The plots present log-transformed PM_2.5_ values for better visualization for high concentrations. Each plot includes a reference line (*y* = *x*) and a fitted regression line to evaluate model performance and bias trends.

The density scatter plot (Figure 2) illustrates log-transformed predictions versus ground truths for the three models, revealing a tendency across all three models to slightly underestimate high concentrations, with fitted line slopes less than 1. Among the models, Bi-LSTM with attention stands out as the top performer, achieving the highest *R*^2^ (0.719; note that this differs from Table 2 due to the log transformation) and the lowest RMSE (0.132), with particularly strong performance at higher concentration levels. The uni-directional (standard) LSTM baseline with attention also performs competitively with an *R*^2^ of 0.689 and an RMSE of 0.139, highlighting the importance of incorporating temporal dependencies. In contrast, the random forest model, which adopts a point-to-point modeling approach, exhibits comparatively lower performance. This evaluation underscores the effectiveness of our Bi-LSTM model with attention in delivering reliable PM_2.5_ predictions.

To further validate the performance of the models across different PM_2.5_ concentration ranges, we categorize the evaluation based on the ground-truth PM_2.5_ level, following established standards [45,46]. The results (shown in Table 3) highlight that Bi-LSTM consistently outperforms the random forest model across all categories. In the “Good” category (<12.0 μg/m^3^), both models tend to overestimate concentrations slightly, with Bi-LSTM achieving a lower RMSE (2.29 μg/m^3^ vs. 2.39 μg/m^3^) and MBE (0.63 μg/m^3^ vs. 0.88 μg/m^3^). However, this overestimation in the “Good” category, which comprises the majority of the dataset, explains why the random forest model achieves a near-zero overall MBE in Table 2, as the overestimations in this dominant category offset the significant underestimations observed in higher pollution ranges. In contrast, Bi-LSTM shows a more balanced behavior, with reduced overestimation in the “Good” category, resulting in a slightly negative overall MBE. As concentrations increase, both models exhibit greater underestimations, but Bi-LSTM demonstrates better robustness with lower RMSE and MBE in each category. Notably, in the most extreme “Hazardous” category (>250.5 μg/m^3^), random forest underestimates by nearly 200 μg/m^3^, while Bi-LSTM reduces this underestimation bias to 126.66 μg/m^3^.

### 3.2. Spatiotemporal Analysis of Bi-LSTM Prediction Bias

To investigate the spatiotemporal patterns of model performance, we divided the CONUS into seven regions based on NCA Climate zones [47] and categorized the seasons into spring (March–May), summer (June–August), autumn (September–November), and winter (December–February). Table 4 presents the seasonal and regional averages of the MBEs for Bi-LSTM predictions of PM2.5. Across all seasons, the northwest exhibits the highest average MBE (0.408), reflecting considerable overestimation in this region, particularly during winter (0.636), likely due to reduced wildfire activity leading to lower PM2.5 concentrations. Conversely, the Northern Great Plains shows the lowest average MBE (0.118), suggesting that the model performs with minimal bias in this region, potentially owing to the relatively uniform pollution levels and the sparse distribution of monitoring stations.

Seasonally, winter displays the highest average MBE (0.356), indicating a tendency for larger biases during this period across most regions. In contrast, autumn has the lowest seasonal average MBE (0.170). These findings align with previous studies [48]. The reduced error in autumn is likely due to the more stable atmospheric conditions and reduced pollution extremes, which increase the predictability of PM_2.5_ [49]. These results demonstrate regional and seasonal variations in model performance, underscoring the importance of developing adaptive strategies to mitigate spatial and temporal biases for more robust PM_2.5_ estimations.

### 3.3. Ablation Study of Wildfire Smoke Density Variable

To demonstrate the impact of including the wildfire smoke density (WSD) variable on the performance of our PM_2.5_ estimation models, we conducted an ablation study shown in Table 5. The table contrasts two versions of our Bi-LSTM model: one without WSD data (“Bi-LSTM w/o WSD”) and one with WSD data (“Bi-LSTM w/ WSD”). The inclusion of WSD data markedly enhances the model’s performance across all metrics. Specifically, the model with WSD variable data exhibits a higher *R*^2^ value of 0.75 compared to 0.72 for the model without WSD, indicating improved variance explanation. Similarly, the RMSE is reduced from 3.78 to 3.59 μg/m^3^, reflecting more accurate predictions. The MBE also shows a lower magnitude of underestimation, improving from −0.27 to −0.08 μg/m^3^. This improvement is even more pronounced in scenarios with PM_2.5_ concentrations above 35 μg/m^3^, where the *R*^2^ improves from 0.41 to 0.44, RMSE decreases from 25.22 to 23.87 μg/m^3^, and MBE shows a significant reduction in underestimation from −11.99 to −9.62 μg/m^3^. These results affirm the significant benefits of integrating WSD data into our model, especially under high pollution conditions, enhancing the estimation precision of PM_2.5_ estimations.

### 3.4. Comparative Analysis with External Dataset

To evaluate the efficacy of our model’s performance for the contiguous United States, we also conducted a comparative analysis with the published dataset by Di et al. [21] (shown in Table 6) and Wei et al. [10] (shown in Table 7). Our findings indicate that our model exhibits superior performance in terms of both RMSE and MBE across both comparisons, especially on high-concentration days.

From 2005 to 2016, our model achieved an average RMSE of 2.63 μg/m^3^, outperforming Di et al. [21]’s ensemble model, which achieved an average RMSE of 2.73 μg/m^3^. Our model showed a small positive MBE of 0.21 μg/m^3^, indicating a slight tendency to overestimate PM_2.5_ concentrations, whereas Di et al. [21]’s model demonstrated a slightly negative MBE of −0.13 μg/m^3^, showing a mild underestimation.

Our model’s performance was particularly robust on high-concentration days (PM_2.5_ levels exceeding 35 μg/m^3^). It achieved an average RMSE of 15.44 μg/m^3^, a substantial improvement compared to Di et al. [21]’s RMSE of 19.01 μg/m^3^. Similarly, the MBE on high-concentration days improved significantly in our dataset, with an average value of −3.41 μg/m^3^ compared to −5.25 μg/m^3^ for the Di et al. [21] dataset. These improvements highlight our model’s capability to reduce bias and provide reliable predictions even during extreme pollution events.

For the post-2016 period (2017–2021), we compared our dataset with Wei et al. [10]’s dataset, as shown in Table 7. Over this period, our model achieved an average RMSE of 2.73 μg/m^3^, significantly lower than Wei et al. [10]’s RMSE of 4.70 μg/m^3^. The bias in our predictions remained low, with an average MBE of 0.24 μg/m^3^ compared to 1.54 μg/m^3^ in Wei et al. [10]’s dataset. Wei et al. [10]’s dataset exhibited a larger RMSE and higher MBE compared to Di et al. [21]’s dataset. Wei et al. [10]’s model designed for global-scale applications likely struggles to capture finer regional variations and adapt to spatial heterogeneity in CONUS.

During high-concentration days, our dataset’s average RMSE was 20.78 μg/m^3^, which is lower than Wei et al. [10]’s average RMSE of 26.45 μg/m^3^. Similarly, the MBE improved from −9.85 μg/m^3^ in Wei et al. [10]’s dataset to −3.34 μg/m^3^ in ours. These results highlight our model’s enhanced ability to predict PM_2.5_ levels during high-pollution periods, even in scenarios with increased wildfire activity post-2016.

The comparative analyses underscore the effectiveness of our Bi-LSTM with attention mechanism in capturing spatiotemporal dependencies and incorporating wildfire smoke density as a predictive variable. The results demonstrate substantial improvements over both the Di et al. [21] and Wei et al. [10] datasets, particularly during high-concentration events. These findings reaffirm the importance of integrating additional environmental variables and leveraging temporal modeling strategies to achieve accurate PM_2.5_ predictions across varying pollution levels.

### 3.5. Case Study of the 2020 California August Complex Fire

**Figure 3.**
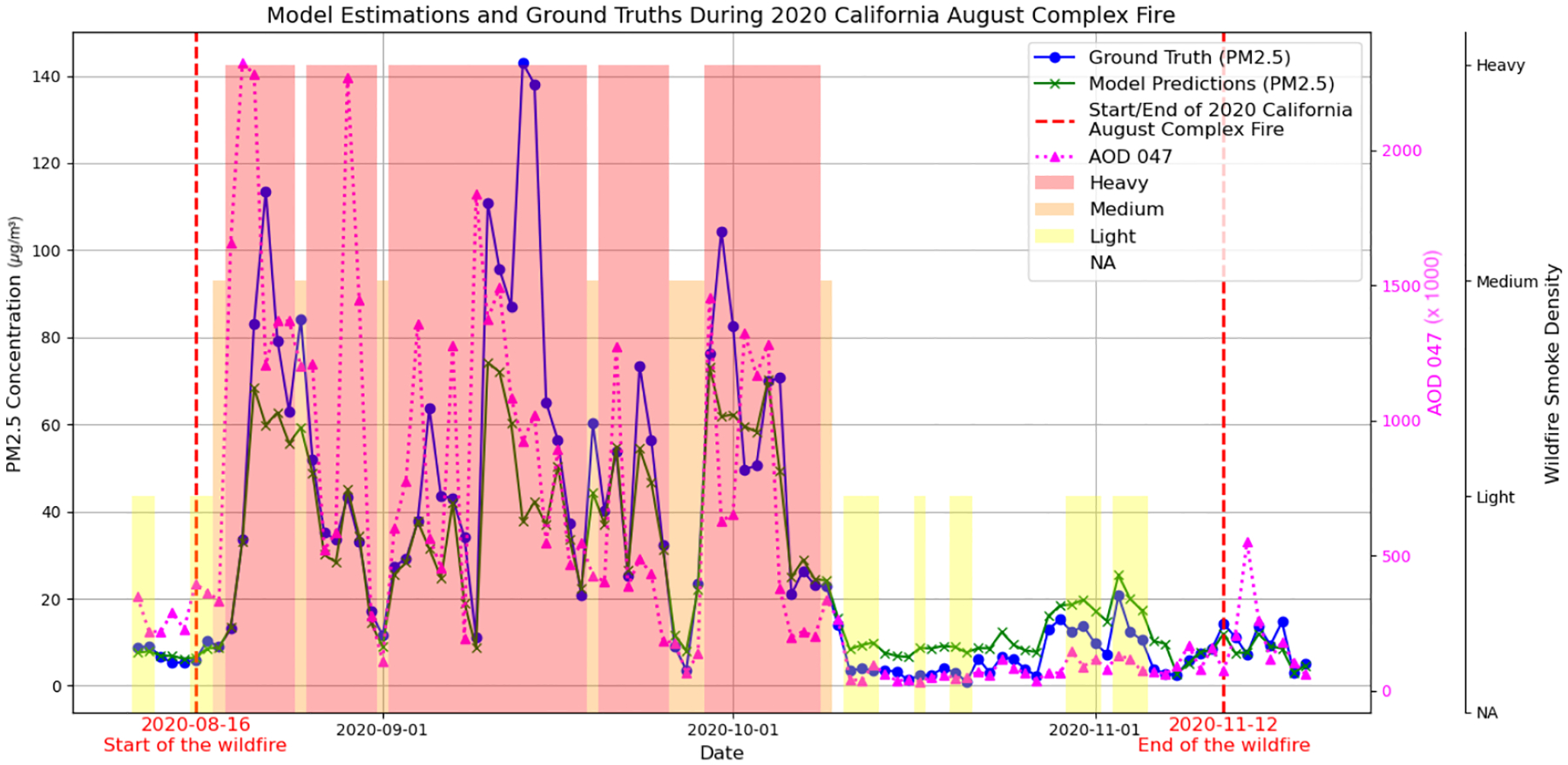
Model estimations and ground truths during the 2020 California August Complex Fire. Blue and green curves represent ground-truth PM_2.5_ and model predictions, respectively. Red dashed lines mark the wildfire start and end dates. The pink curve shows AOD 047, while shaded areas indicate wildfire smoke density, with darker shades representing higher severity.

Figure 3 demonstrates that increased wildfire smoke density corresponds with spikes in measured PM_2.5_ levels. The figure demonstrates ground-truth measured PM_2.5_ levels (blue curve) against our model’s predictions (green curve), alongside the brown curve representing the AOD 047 band values for additional context. The shaded backgrounds—ranging from yellow to red—denote varying intensities of wildfire smoke density, segmented into light, medium, and heavy categories, which visually correlate with observed fluctuations in PM_2.5_ values.

Figure 3 underscores periods where the smoke density is heightened, aligning with spikes in both AOD and PM_2.5_ readings. Notably, our model closely mirrors the ground truth values in lower smoke density scenarios but exhibits some deviation under heavier smoke conditions, suggesting areas for further refinement in smoke-impacted PM_2.5_ prediction. While estimating extreme values is a challenge for machine learning regression in general [50,51], our Bi-LSTM improves estimations on high-concentration days, as can be seen in Tables 6 and 7. However, there is still noticeable underestimation. Another potential reason for this underestimation under heavy smoke conditions is that all high concentrations are classified under the single category of ‘heavy’ smoke, regardless of intensity, which limits the model’s ability to distinguish more granular PM_2.5_ levels within this range. Nevertheless, our model’s ability to better capture the impact of smoke density compared to previous methods addresses a gap that has not been directly addressed in earlier modeling efforts [21,24].

**Figure 4.**
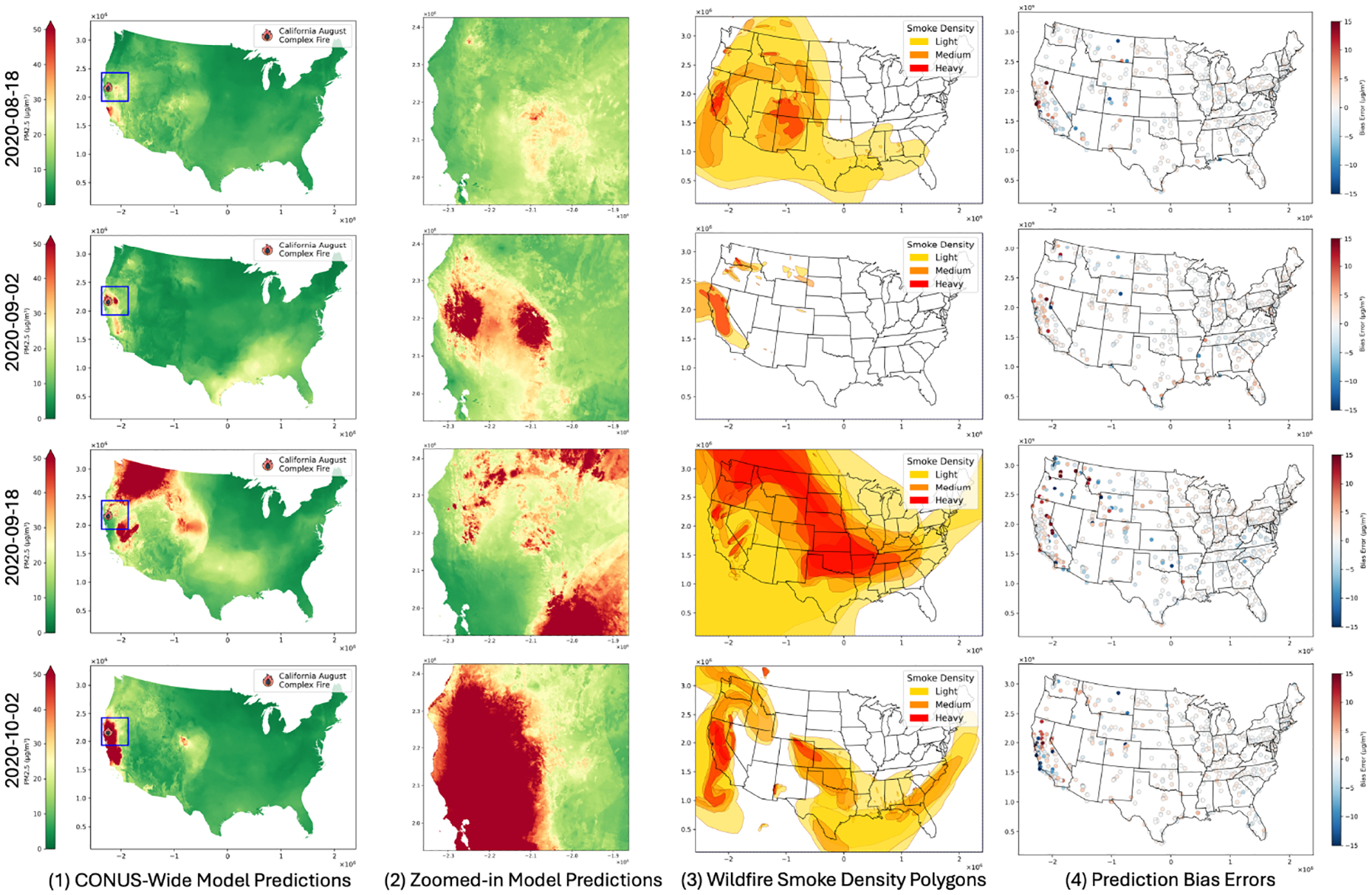
Spatial and temporal analysis of PM_2.5_ predictions during the 2020 California August Complex Fire. The columns represent (1) CONUS-wide model predictions; (2) Zoomed-in predictions of the wildfire region (sharing the same colorbar as (1), ranging from 0 to 50 μg/m^3^); (3) HMS wildfire smoke density polygons; and (4) prediction bias errors. Rows correspond to four dates during different wildfire phases: ignition (18 August 2020), spreading (2 September 2020), intensification (18 September 2020), and later progression (2 October 2020).

Expanding the temporal analysis to a spatial context, Figure 4 provides CONUS-wide and zoomed-in views of PM_2.5_ predictions during four different phases of the wildfire-initial ignition (18 August 2020), spreading (2 September 2020), intensification (18 September 2020), and later progression (2 October 2020). The spatial prediction maps (first column) depict the large-scale dispersion of PM_2.5_, while the zoomed-in maps (second column) provide a closer look at the wildfire-affected regions, highlighting the progression from localized areas of moderate concentrations to widespread regions with significantly elevated PM_2.5_ levels over time. Wildfire smoke density polygons (third column) further strengthen these findings, highlighting regions of heavy smoke that align with elevated PM_2.5_ predictions. However, the prediction bias error maps (fourth column) reveal significant spatial heterogeneity, particularly in the Western U.S., where biases are generally higher than in the east. These spatial patterns highlight the challenges of accurately capturing PM_2.5_ levels during wildfires, emphasizing the need for continuing refinement in modeling localized smoke dispersion and atmospheric processes.

## 4. Discussion

### 4.1. Advantages of Temporal Modeling

Our results demonstrate the effectiveness of temporal deep learning techniques leveraging spatial features against traditional point-to-point strategies in predicting PM_2.5_ concentrations. Point-to-point models cannot tap into the crucial temporal information that is common in environmental measurements. These models treat observations as independent events, which is suboptimal in the context of the dynamic and continuous nature of atmospheric conditions that influence air quality. On the other hand, our Bi-LSTM with attention architecture demonstrates improvement in capturing these dependencies. LSTMs effectively utilize historical data, learning from past trends and patterns to forecast future states, and Bi-LSTMs leverage observed pre- and post-event observations for further enhancement. For instance, the buildup of pollutants over time or the progression of a wildfire can be critical for accurate predictions, intuitions that point-to-point models typically miss. Moreover, our results indicate that temporal models are not only better at handling time-series data but also improve prediction reliability during critical events such as wildfires. By integrating both past data and spatial interactions (e.g., our KNN-IDW-derived features), these models provide a more holistic picture of environmental phenomena, leading to more precise estimations.

### 4.2. Computational Trade-Offs and Model Efficiency

Random forest requires approximately 1.5 h to train on 3,012,648 samples using a 20-core CPU (Intel Xeon W-1290P), while Bi-LSTM training takes about 2.5 h on an NVIDIA RTX 3080 GPU. For inference, random forest completes predictions for PM_2.5_ across the CONUS in 3 min, compared to 7 min for Bi-LSTM. The higher computational cost of Bi-LSTM arises from its 21-day time-series inputs, which enable the model to capture temporal dependencies, whereas a random forest processes single-day inputs. However, the superior predictive performance of Bi-LSTM justifies the increased computational costs, particularly for applications requiring higher accuracy in air pollution estimations.

### 4.3. Limitations and Future Research

There are limitations associated with the quality of HMS smoke density variable. Notably, the dataset exhibits gaps in daily measurements, with certain years missing several days worth of data. Furthermore, the classification into smoke density categories—light, medium, and heavy—is missing in the earlier years (2005–2007, parts of 2008 and 2010), and some wildfire smoke events may not be captured [52]. These discrepancies may lead to underestimations or missed detections of smoke’s impact on surface air quality, especially since the HMS smoke product primarily represents smoke as observed from satellites. As highlighted by Liu et al. [53], the satellite imagery may depict smoke that is aloft, and thus not affecting ground-level air quality, particularly in the case of light smoke. The dataset also suffers from reduced spatial coverage in earlier years and other caveats, such as the reliance on daytime satellite imagery for smoke detection and the occasional presence of poorly configured smoke polygons that result in data errors [53]. Given these limitations, it is advisable to treat trends in smoke data with caution, particularly outside the contiguous United States. To overcome some of these challenges, future studies should explore the use of Liu et al. [53]’s imputed smoke density datasets, which may provide more coverage with the representation of surface smoke presence and its duration.

Another limitation stems from the reporting frequency of AQS PM_2.5_ monitoring stations, though year-over-year improvement is evident over the span from 2005 (67% daily measurements) to 2021 (96% daily measurements). Despite these improvements, the efficacy of our temporal model, which integrates spatial features using inverse distance weighting (IDW), is hampered by irregular reporting intervals. While IDW is a straightforward approach for incorporating spatial data, it may oversimplify spatial dependencies and overlook spatial heterogeneity, especially in areas with sparse or unevenly distributed monitoring stations. Recent advancements in graph neural networks (GNNs) offer more sophisticated methods for capturing spatial relationships and represent a promising direction for integrating ground-based monitoring data into future models [54]. The irregular intervals often extend from three to six days without reported values, undermining the consistency required for a temporal model to fully leverage temporal dynamics. This issue deteriorates performance during critical environmental incidents, such as periods of heightened pollution resulting from wildfires, when it is even more important to accurately characterize exposure to air pollution.

Furthermore, while our Bi-LSTM model excels in capturing temporal dependencies, it is less effective at leveraging local spatial patterns inherent in raster data, such as satellite-derived AOD and meteorological reanalysis data. Convolutional neural networks (CNNs), known for their ability to extract spatial features from gridded data, could complement the current framework to better address this limitation and improve the model’s ability to incorporate spatial information [54,55]. Future research should explore hybrid models that integrate temporal and spatial components, such as CNN-LSTM architectures, to enhance accuracy in areas with complex spatial and temporal dynamics. Additionally, models capable of accommodating and adapting to irregular time-series data could further improve performance and robustness in capturing air quality trends.

Deep learning models are often critiqued for their lack of interpretability and explainability. While attention weights offer insights into temporal dynamics, their interpretation must be approached with caution, as they can be sometimes misleading [56]. Investigating the temporal lag effects of PM_2.5_ dispersion through more robust analyses of attention mechanisms could provide valuable insights. Understanding feature importance is particularly critical for policymaking. Future work could explore advanced interpretability techniques, such as permutation importance [57], SHAP (SHapley Additive exPlanations) [58], and GeoSHAP [59], to provide a more comprehensive understanding of feature contributions.

## 5. Conclusions

We presented an approach for estimating daily PM_2.5_ surface-level concentrations by integrating satellite observations, auxiliary variables, and ground measurements using a Bi-LSTM with attention architecture. Our evaluations demonstrated the superior performance of our temporal modeling approach over the widely used tree-based ensemble baseline and the existing dataset by Di et al. [21] in predicting PM_2.5_ concentrations, especially on high-concentration days. This significant improvement is largely attributed to the integration of our model, which enhances its ability to predict high PM_2.5_ concentrations during wildfire events, aligning closely with observed spikes and, thus, addressing a crucial gap in previous modeling efforts. Our work not only contributes to air pollution modeling literature by reducing estimation error, but the resulting products and open-source code enable and improve follow-up research relying on such products. The daily 1 km product allows quantifying cohort subjects’ exposure to PM_2.5_ in longitudinal studies and, consequently, supports effective public health policy-making and interventions.

Our analysis highlights the pivotal role of AOD as a predictive variable, though its limitations in capturing rapid air quality fluctuations during intense wildfire episodes suggest a need for the incorporation of more dynamic data sources, such as real-time smoke measurements. NASA’s recent deployment of the Tropospheric Emissions: Monitoring of Pollution (TEMPO) instrument could significantly enhance air pollution modeling efforts by providing higher spatial resolution and hourly updates on air quality factors, from rush-hour traffic to forest fires, across North America [60].

The inconsistencies and temporal gaps in measurements such as those seen in the HMS smoke density category levels, however, could negatively impact estimation performance. Moreover, in some cases, the irregular reporting frequency of PM_2.5_ monitoring stations poses challenges in modeling efforts, particularly during critical pollution events. Future research should focus on refining data imputation techniques and enhancing model adaptability to leverage irregular temporal inputs, thus improving the predictive performance and reliability of air quality models.

## Figures and Tables

**Table 1.** Predictor variables.

Variables	Source	Spatial Resolution	Temporal Resolution	Number of Variables
Satellite-derived AOD of Blue (0.47 μm) and Green (0.55 μm) Band.	MODIS MCD19A2.061	1 km	Daily	2
Meteorological conditions: Day Length (dayl), Precipitation (prcp), Shortwave Radiation (srad), Maximum Air Temperature (tmax), Minimum Air Temperature (tmin), and Water Vapor Pressure (vp).	Daymet	1 km	Daily	6
Meteorological conditions: Wind Direction (th), and Wind Velocity (vs)	gridMET	~4 km, 1/24th degree	Daily	2
Wildfire Smoke Density (WSD).	NOAA’s Hazard Mapping System (HMS) Smoke Product	Polygon	Daily	1
Elevation.	GMTED2010	1 km	NA	1
Combined 16-Day NDVI.	MODIS MCD43A4	500 m	16-Day	1
Inverse Distance Weighted PM_2.5_.		NA	Daily	1
Spatial Features: Latitude and Longitude.		NA	NA	2
Temporal Encodings: Cos/Sin(Day of the Year, Month of the Year), and Year.		NA	Daily	5

**Table 2.** The 10-fold random cross-validation and testing results of PM_2.5_ estimation.

Model	Cross-Validation			Testing		
	*R* ^2^	RMSE [μg/m^3^]	MBE [μg/m^3^]	*R* ^2^	RMSE [μg/m^3^]	MBE [μg/m^3^]
Random Forest	0.69	3.98	0.00	0.68	4.00	0.00
LSTM	0.72	3.77	−0.11	0.72	3.81	−0.10
Bi-LSTM (ours)	0.74	3.67	−0.09	0.75	3.59	−0.08

**Table 3.** Model performance evaluation by PM_2.5_ categories.

Category (PM_2.5_ Range [μg/m^3^])	Num. Samples	Random Forest		Bi-LSTM	
		RMSE [μg/m^3^]	MBE [μg/m^3^]	RMSE [μg/m^3^]	MBE [μg/m^3^]
Good (0.0–12.0)	2,329,390	2.39	0.88	2.29	0.63
Moderate (12.1–35.4)	629,680	4.93	−2.63	4.58	−1.76
Unhealthy for Sensitive Groups (35.5–55.4)	15,850	18.00	−13.70	14.50	−8.17
Unhealthy (55.5–150.4)	4830	36.93	−26.62	32.83	−16.91
Very Unhealthy (150.5–250.4)	250	111.25	−102.59	85.32	−67.68
Hazardous (250.5–∞)	90	216.36	−190.13	156.76	−126.66

**Table 4.** Seasonal and regional average mean bias error (MBE) of Bi-LSTM predictions for PM_2.5_.

Region	Spring	Summer	Autumn	Winter	Avg
Midwest	0.248	0.249	0.187	0.355	**0.260**
Northeast	0.159	0.177	0.143	0.243	**0.181**
Northern Great Plains	0.075	0.084	0.114	0.200	**0.118**
Northwest	0.430	0.200	0.367	0.636	**0.408**
Southeast	0.138	0.167	0.120	0.180	**0.151**
Southern Great Plains	0.086	0.246	0.160	0.219	**0.178**
Southwest	0.315	0.175	0.097	0.656	**0.311**
**Avg**	**0.207**	**0.185**	**0.170**	**0.356**	–

**Table 5.** Ablation study results comparing model performance with and without wildfire smoke density (WSD) variable.

Model	*R* ^2^	RMSE [μg/m^3^]	MBE [μg/m^3^]	*R*^2^ (>35 μg/m^3^) [μg/m^3^]	RMSE (>35 μg/m^3^) [μg/m^3^]	MBE (>35 μg/m^3^) [μg/m^3^]
Bi-LSTM w/o WSD	0.72	3.78	−0.27	0.41	25.22	−11.99
Bi-LSTM w/ WSD	0.75	3.59	−0.08	0.44	23.87	−9.62

**Table 6.** Comparison of PM_2.5_ estimation performance between our dataset and Di et al. [21]’s (2019) baseline during the 2005 to 2016 period common between the two datasets.

	Di et al. [21] (2019) Dataset				Our Dataset			
Year	RMSE [μg/m^3^]	MBE [μg/m^3^]	RMSE (>35 μg/m^3^) [μg/m^3^]	MBE (>35 μg/m^3^) [μg/m^3^]	RMSE [μg/m^3^]	MBE [μg/m^3^]	RMSE (>35 μg/m^3^) [μg/m^3^]	MBE (>35 μg/m^3^) [μg/m^3^]
2005	2.82	−0.07	10.18	−2.76	2.90	0.15	8.67	−2.40
2006	2.51	−0.19	12.73	−3.23	2.69	0.15	12.15	−3.61
2007	2.86	−0.17	14.62	−5.88	2.87	0.19	12.04	−3.29
2008	2.56	−0.21	16.36	−7.28	2.56	0.18	13.74	−4.00
2009	2.63	−0.07	19.14	−4.97	2.62	0.19	16.52	−3.05
2010	2.47	−0.03	15.47	−3.62	2.51	0.23	14.39	−4.32
2011	2.56	−0.08	16.35	−4.84	2.67	0.28	15.42	−3.08
2012	2.86	−0.11	26.76	−8.03	2.76	0.25	21.98	−2.21
2013	2.80	−0.07	19.94	−2.76	2.62	0.21	16.17	−0.83
2014	2.53	−0.16	20.48	−2.06	2.43	0.13	15.50	−4.43
2015	2.60	−0.11	22.24	−1.57	2.54	0.26	18.62	−3.75
2016	3.54	−0.25	33.83	−16.07	2.37	0.29	20.06	−5.97
**Avg**.	**2.73**	−**0.13**	**19.01**	−**5.25**	**2.63**	**0.21**	**15.44**	−**3.41**

**Table 7.** Comparison of PM_2.5_ estimation performance between our dataset and Wei et al. [10]’s (2023) during the 2017 to 2021 period common between the two datasets.

	Wei et al. [10] (2023) Dataset				Our Dataset			
Year	RMSE [μg/m^3^]	MBE [μg/m^3^]	RMSE (>35 μg/m^3^) [μg/m^3^]	MBE (>35 μg/m^3^) [μg/m^3^]	RMSE [μg/m^3^]	MBE [μg/m^3^]	RMSE (>35 μg/m^3^) [μg/m^3^]	MBE (>35 μg/m^3^) [μg/m^3^]
2017	5.55	2.28	26.95	−9.15	2.58	0.21	18.40	−1.20
2018	4.92	1.97	21.43	−6.61	2.66	0.23	20.12	−2.28
2019	3.37	1.25	20.49	−14.08	1.96	0.25	16.19	−4.20
2020	5.31	1.04	39.10	−10.13	3.49	0.25	27.52	−1.59
2021	4.36	1.17	24.26	−9.27	3.17	0.21	23.88	−6.14
**Avg**.	**4.70**	**1.54**	**26.45**	−**9.85**	**2.73**	**0.24**	**20.78**	−**3.34**

## Data Availability

The PM_2.5_ estimation dataset and codes used in this study are freely available as a public resource via the GeoHAI website at https://geohai.org/projects/estimating-air-pollution.html (accessed on 30 December 2024) or by contacting the authors.

## Appendix A. AOD Imputation Methods and Results

Aerosol Optical Depth (AOD) is a critical predictor in PM_2.5_ estimation; however, its coverage is frequently limited by factors such as cloud cover, orbit patterns, and the presence of snow or bright surfaces. Additionally, while our PM_2.5_ model employs a masking layer to manage missing values, utilizing a dedicated imputation model can further refine performance and alleviate the computational burden on the primary model. For this present study, we developed an AOD imputation methodology based on our previous imputation work [61]; however, instead of random forests, here we leverage XGBoost [62] and incorporate mean filters, resulting in enhanced spatial accuracy. Mean filters are widely used in image processing to smooth variations between adjacent pixels and reduce noise. This approach not only expands the spatial coverage of AOD data but also introduces a spatial prior that markedly boosts model performance. The effectiveness of these methodological improvements is evidenced in Table A1, showcasing enhanced performance for imputing AOD. Specifically, for the AOD 047 band without mean filters, our imputation approach dramatically reduced the validation RMSE to 82.08 from random forest’s 101.52 baseline, albeit with a minor increase in MBE of 0.27. For the AOD 055 band, with mean filters applied, our imputation decreased the RMSE to 60.63 from 75.13 and changed the slight MBE from −0.16 to 0.08. These outcomes underline the superior performance of XGBoost with mean filter for AOD imputation, advancing our ability to use imputed AOD values as input in our final model to estimate surface-level PM_2.5_ concentrations.

**Table A1.** The 10-fold random cross-validation results of AOD imputation.

	Random Forest		XGBoost	
	Val RMSE	Val MBE	Val RMSE	Val MBE
AOD 047 with mean filters	31.75 ± 0.81	−0.02 ± 0.10	36.36 ± 0.74	0.01 ± 0.10
AOD 047 w/o mean filters	101.52 ± 1.22	−0.02 ± 0.33	82.08 ± 0.96	0.27 ± 0.34
AOD 055 with mean filters	23.12 ± 0.43	−0.01 ± 0.08	26.64 ± 0.47	0.00 ± 0.07
AOD 055 w/o mean filters	75.13 ± 0.88	−0.16 ± 0.24	60.63 ± 1.02	0.08 ± 0.14

## Abbreviations

The following abbreviations are used in this manuscript:

LSTM: Long Short-Term Memory
PM: Particulate Matter
LUR: Land Use Regression
MODIS: Moderate Resolution Imaging Spectroradiomete
AOD: Aerosol Optical Depth
MAIAC: Multi-Angle Implementation of Atmospheric Correction
EPA: Environmental Protection Agency
AQS: Air Quality System
NOAA: National Oceanic and Atmospheric Administration
HMS: Hazard Mapping System
NDVI: Normalized Difference Vegetation Index
KNN: K-Nearest Neighbor
IDW: Inverse Distance Weighting
Bi-LSTM: Bidirectional Long Short-Term Memory
RNN: Recurrent Neural Networks
RMSE: Root Mean Squared Error
MBE: Mean Bias Error
WSD: Wildfire Smoke Density
TEMPO: Tropospheric Emissions: Monitoring of Pollution
